# Cundurango

**Published:** 1871

**Authors:** 


### *Cundurango.*

Within the past few weeks we have received letters from Medical friends, in different parts of the State, asking reliable information relative to the remarkable virtues claimed for this South American remedy for Cancer.

We very much regret our inability, at present, to give the desired information, but fear, from the conflicting reports of gentlemen who have had opportunities of testing its virtues, that it will ultimately be added to that numerous class of agents which have, from time to time, agitated the public mind and disappointed the hopes of so many unfortunate victims of this terrible malady.

That our readers may keep pace with the status of this investigation, we publish in this number of the Journal a communication from Dr. Bliss, of Washington, D. C., to the New York Medical Journal, in which he gives the result of his experience with the cundurango in the treatment of cancer, which he claims has been of the most satisfactory character.

Did the results reported by other medical gentlemen who have had the same opportunities of testing its virtues confirm Dr. Bliss' statements, we would have no longer any reason to doubt the efficacy of this agent in the treatment of cancer.

We are sorry to say, however, that such is not the case, but on the contrary, are in direct conflict with Dr. Bliss' deductions, as will be seen by the following extracts from a communication in the July number of the Richmond and Louisville Medical Journal, by Alex. Y. A. Garnett, M. D., of Washington, D. C.

After alluding to the history of this drug, its chemical analysis, &c., Dr. Garnett says:

"Through the courtesy of Surgeon Norris, of the United States Army, I have been permitted to read his report, made to Surgeon General Barnes, detailing the results of his experience with the cundurango in the treatment of cancer, and, although not authorized to de so by publication, I shall take the liberty of stating that, although the case selected for experiment by him presented all the conditions required for a fair test of its merits, it utterly failed to arrest the progress of the disease, or in any decided manner to modify its character or mitigate the suffering of the patient. The case terminated fatally at the expiratian of five weeks from the commencement of this treatment, notwithstanding its uninterrupted administration during that period. I will here add, that exactly similar results followed in another case which was treated in New York with the cundurango, under instructions from the Surgeon General."

After mentioning two other cases of cancer, treated with cundurango by a medical officer of the army in Washington City, with no favorable results, he concludes his communication with the following tart rebuke:

"In conclusion, I would simply remark that the apparent and conceded attempt to manufacture a fictitious reputation for this drug, resting as it does upon such questionable data, seems rather to have been inspired by the dazzling prospect of pecuniary speculation, than by a laudable desire to advance the triumphs of science, or to alleviate the sufferings of unfortunate humanity."

It is evident that too little is known of this remedy to warrant the expression of an opinion, and as unfair dealing is hinted at, it is necessary to wait further developments.

